# An Updated Systematic Review and Meta-Analysis of the Association between the De Ritis Ratio and Disease Severity and Mortality in Patients with COVID-19

**DOI:** 10.3390/life13061324

**Published:** 2023-06-05

**Authors:** Arduino A. Mangoni, Angelo Zinellu

**Affiliations:** 1 Discipline of Clinical Pharmacology, College of Medicine and Public Health, Flinders University, Bedford Park, SA 5042, Australia; 2 Department of Clinical Pharmacology, Flinders Medical Centre, Southern Adelaide Local Health Network, Bedford Park, SA 5042, Australia; 3 Department of Biomedical Sciences, University of Sassari, 07100 Sassari, Italy; azinellu@uniss.it

**Keywords:** De Ritis ratio, aspartate transaminase, alanine transaminase, COVID-19, disease severity, mortality, biomarkers

## Abstract

Patients with Coronavirus disease 2019 (COVID-19) often have elevations in markers of liver injury, particularly serum aspartate transaminase (AST) and alanine transaminase (ALT). Such alterations may affect the AST/ALT ratio (De Ritis ratio) and, potentially, clinical outcomes. We conducted an updated systematic review and meta-analysis of the association between the De Ritis ratio and COVID-19 severity and mortality in hospitalized patients. PubMed, Web of Science, and Scopus were searched between 1 December 2019 and 15 February 2023. The Joanna Briggs Institute Critical Appraisal Checklist and the Grading of Recommendations, Assessment, Development, and Evaluation were used to assess the risk of bias and the certainty of the evidence, respectively. Twenty-four studies were identified. The De Ritis ratio on admission was significantly higher in patients with severe disease and non-survivors vs. patients with non-severe disease and survivors (15 studies, weighted mean difference = 0.36, 95% CI 0.24 to 0.49, *p* < 0.001). The De Ritis ratio was also associated with severe disease and/or mortality using odds ratios (1.83, 95% CI 1.40 to 2.39, *p* ˂ 0.001; nine studies). Similar results were observed using hazard ratios (2.36, 95% CI 1.17 to 4.79, *p* = 0.017; five studies). In six studies, the pooled area under the receiver operating characteristic curve was 0.677 (95% CI 0.612 to 0.743). In our systematic review and meta-analysis, higher De Ritis ratios were significantly associated with severe disease and mortality in COVID-19 patients. Therefore, the De Ritis ratio can be useful for early risk stratification and management in this patient group (PROSPERO registration number: CRD42023406916).

## 1. Introduction

Coronavirus disease 2019 (COVID-19), an infection caused by the severe acute respiratory syndrome coronavirus 2 (SARS-CoV-2), has been declared a global health emergency by the World Health Organization since 2020 [1]. According to recent estimates, at least 15 million people have died as a result of COVID-19 [2]. However, the long-term clinical and psychological impact of the disease on survivors remain to be fully established [3,4]. Despite significant advances in the prevention and management of COVID-19, particularly with the introduction of vaccination programs at the population level and several effective antiviral and anti-inflammatory treatments, a significant number of patients are still at risk of severe disease and adverse outcomes, including death [5]. One important factor responsible for the ongoing public health burden of COVID-19 is the systemic, multi-organ involvement during the acute phase following infection [6,7]. The onset and the progression of multi-organ dysfunction, particularly in severe cases, is primarily driven by the excessive systemic activation of pro-inflammatory pathways that is accompanied by the release of several cytokines with toxic effects on cellular, tissue, and organ homeostatic mechanisms [8,9]. A critical organ affected by this systemic pro-inflammatory state is the liver. Manifestations of liver injury range from transient elevations of liver enzymes and alterations in specific markers of inflammation and coagulation to severe liver injury and acute liver failure [10,11,12,13,14]. Studies have suggested that the pathogenesis of liver injury involves several mechanisms, including the direct cytotoxic effects of SARS-CoV-2 on hepatocytes and the onset of immune-mediated hepatitis, hypoxic injury, and liver toxicity induced by specific drugs used to treat COVID-19 [15,16,17,18].

Elevations of circulating markers of liver injury in the context of COVID-19, particularly serum aspartate transaminase (AST) and alanine transaminase (ALT), are associated with severe disease and adverse outcomes, including death [19,20,21,22,23,24,25,26,27]. A derived parameter, the AST/ALT ratio (De Ritis ratio), has also been associated with COVID-19 severity and mortality [28,29]. Notably, the capacity of the De Ritis ratio to discriminate between non-severe vs. severe disease and survivor vs. non-survivor status has been shown to be superior to that of AST or ALT alone, further supporting the potential utility of the De Ritis ratio to stratify risk in patients with COVID-19 [30,31,32,33,34].

A systematic review and meta-analysis of eight studies published in 2021 reported that relatively higher De Ritis ratios were significantly associated with poor prognosis in COVID-19 patients, defined as a composite of death, clinical severity, admission to the intensive care unit (ICU), and intubation [35]. Given the rapidly evolving field and clinical scenario with the occurrence of new variants of SARS-CoV-2, several studies investigating the De Ritis ratio in COVID-19 patients have been published since this meta-analysis. Therefore, we conducted an updated systematic review and meta-analysis of the association between this biomarker and COVID-19, investigating a wide range of effect measures as well as prognostic capacity.

## 2. Methods

### 2.1. Systematic Literature Search

A systematic search was conducted in the electronic databases Scopus, Web of Science, and PubMed, between 1 December 2019 and 15 February 2023, using the following terms: “De Ritis ratio” or “AST ALT ratio” or “glutamic oxaloacetic transaminase (GOT) glutamic pyruvic transaminase (GPT) ratio” or “alanine aminotransferase aspartate aminotransferase ratio” and “COVID-19” or “2019-nCoV” or “SARS-CoV-2” or “coronavirus disease 2019”. The reference lists of each article were also searched to identify other studies.

We included studies that: (a) investigated COVID-19 patients with different disease severity or survival status; (b) reported De Ritis ratios as continuous data, odds ratios (ORs), or hazard ratios (HRs) with 95% confidence intervals (CIs) for clinical outcomes using multivariate analysis; (c) reported prognostic accuracy using the area under the receiver operating characteristic curve (AUROC) with 95% CIs; (d) were available in full-text; and (e) used the English language. Two investigators independently reviewed abstracts and articles, with a third involved if there was any disagreement.

The extracted data from each study included study country, year of study publication, study design, age, the proportion of males, sample size, measures of disease severity and survival status, white blood cell count (WBC), C-reactive protein (CRP), D-dimer, history of diabetes, hypertension, cardiovascular disease, mean De Ritis ratio, and OR, HR, and AUROC with 95% CIs, cut-off values, sensitivity, and specificity. We assessed the risk of bias using the Joanna Briggs Institute Critical Appraisal Checklist for case-control studies. Studies addressing ≥75% of items were classified as low risk [36]. We assessed the certainty of evidence using the Grading of Recommendations, Assessment, Development, and Evaluation (GRADE) [37]. The study complied with the Preferred Reporting Items for Systematic Reviews and Meta-Analyses (PRISMA) 2020 statement (Supplementary Tables S1 and S2) [38]. The study protocol was registered in the International Prospective Register of Systematic Reviews (PROSPERO, CRD42023406916).

### 2.2. Statistical Analysis

We generated forest plots from weighted mean differences (WMDs) to assess the De Ritis ratio in patients with non-severe disease or survivor status and patients with severe disease or non-survivor status (*p* < 0.05 for statistical significance). Adjusted ORs or HRs were transformed into log ORs and log HRs, and the standard error was calculated based on the corresponding 95% CI. Heterogeneity was assessed using the Q statistic (*p* < 0.10 for statistical significance). We used a random-effect model in case of moderate–substantial heterogeneity (I^2^ values ≥ 30%) [39]. We assessed the influence of individual studies through sensitivity analysis [40]. Publication bias was assessed with the Begg’s and Egger’s tests (*p* < 0.05 for statistical significance) and the Duval and Tweedie “trim-and-fill” procedure [41,42,43]. In univariate meta-regression analysis, we investigated associations between the WMD/OR/HR and participant age, sex, study sample size, year of study publication, study design, markers of inflammation (WBC, CRP), markers of coagulation (D-dimer), and a history of diabetes, hypertension, and cardiovascular disease. In subgroup analyses, we investigated possible differences in effect size according to the clinical outcome studied (disease severity vs. mortality) and study continent. The prognostic performance of the De Ritis ratio was assessed by calculating the weighted summary AUROC using the fixed and/or random effects model [44]. Stata 14 software was used for statistical analysis (StataCorp LLC, College Station, TX, USA).

## 3. Results

### 3.1. Selection of Studies

**Figure 1:**
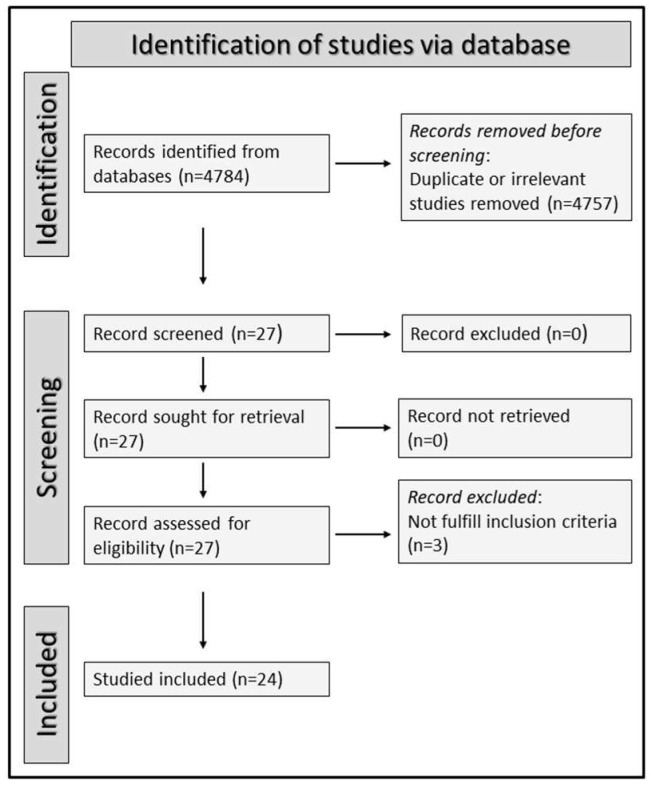
PRISMA 2020 flow chart.

A total of 4784 articles were initially identified. Of them, 4757 were either duplicates or irrelevant and, therefore, excluded. After a full review of the remaining 27 articles, three were excluded because they did not meet the inclusion criteria, leaving 24 articles for analysis (Figure 1) [30,31,32,33,34,45,46,47,48,49,50,51,52,53,54,55,56,57,58,59,60,61,62,63].

Two studies had a prospective study design [45,53], whilst the remaining 22 were retrospective [30,31,32,33,34,46,47,48,49,50,51,52,54,55,56,57,58,59,60,61,62,63]. The clinical endpoints assessed included mortality (15 studies) [30,31,32,33,34,46,47,48,50,51,52,54,55,56,62] and the following measures of severe disease: clinical severity based on existing guidelines (8 studies) [33,45,49,53,56,57,58,59], transfer to the ICU (two studies) [56,62], persistent viral positivity (one study) [61], prolonged hospital stay (one study) [62], intubation (one study) [63], and a composite endpoint of mortality or ICU transfer (one study) [60]. The De Ritis ratio was assessed within the first 24–48 h from hospital admission in all studies. The observational design in all studies was the primary reason for initially grading the initial level of certainty as low (rating 2).

### 3.2. Pooled Mean Differences

#### 3.2.1. Characteristics of Studies

Fifteen studies reported De Ritis ratios in 5923 COVID-19 patients (mean age 69 years, 59% males) with non-severe disease or survivor status and 1898 patients (mean age 56 years, 55% males) with severe disease or non-survivor status [30,31,34,45,46,47,48,49,50,52,53,57,58,59,60]. Four studies were conducted in China [45,49,53,59], two in Italy [31,34], one in Spain [48], one in Nepal [57], one in India [58], one in Mexico [60], one in Austria [46], one in Korea [47], one in Romania [50], and one in Hungary [52]. Clinical endpoints included mortality (eight studies) [30,31,34,46,47,48,50,52] and measures of disease severity in the remaining seven (Supplementary Table S3) [45,49,53,57,58,59,60].

#### 3.2.2. Risk of Bias

All studies addressed ≥75% of checklist items and, therefore, had a low risk of bias (Supplementary Table S4) [30,31,34,45,46,47,48,49,50,52,53,57,58,59,60].

#### 3.2.3. Results of Individual Studies and Syntheses

**Figure 2:**
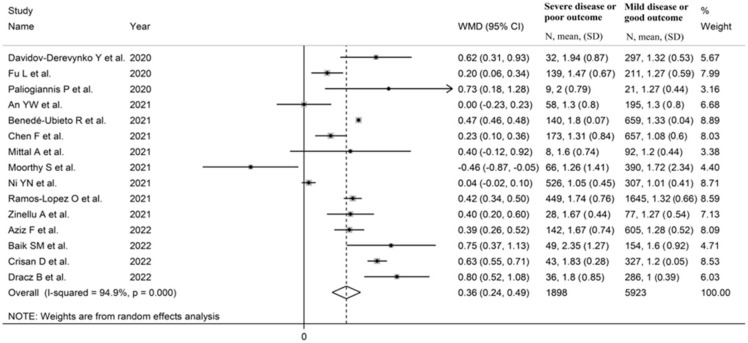
Forest plot of studies investigating differences in De Ritis ratio between patients with non-severe disease or survivor status and patients with severe disease or non-survivor status.

The forest plot of De Ritis ratios in patients with non-severe vs. severe disease or survivor vs. non-survivor status is shown in Figure 2. Random-effects models were used given the moderate–substantial heterogeneity observed (I^2^ = 94.9%, *p* < 0.001). Pooled results showed that the De Ritis ratios were significantly higher in patients with severe disease or non-survivor status (WMD = 0.36, 95% CI 0.24 to 0.49, *p* < 0.001). The sequential removal of individual studies did not substantially alter the corresponding pooled WMD values (range 0.34–0.40).

#### 3.2.4. Publication Bias

There was no publication bias according to the Begg’s (*p* = 1.00) or the Egger’s (*p* = 0.25) test. Accordingly, no missing study to be added to the funnel plot was identified using the “trim-and-fill” method (Supplementary Figure S1).

#### 3.2.5. Subgroup and Meta-Regression Analysis

In meta-regression, the WMD was not significantly associated with age (t = −0.26, *p* = 0.808), the proportion of males (t = −0.90, *p* = 0.39), publication year (t = 1.01, *p* = 0.33), study design (t = 1.38, *p* = 0.19), sample size (t = −0.40, *p* = 0.69), WBC (t = 0.79, *p* = 0.45), CRP (t = −1.62, *p* = 0.14), D-dimer (t = 0.71, *p* = 0.50), or history of diabetes (t = −0.84, *p* = 0.43), hypertension (t = −0.39, *p* = 0.71), and cardiovascular disease (t = −0.11, *p* = 0.92). In subgroup analysis, the pooled WMD in studies investigating disease severity (WMD = 0.09, 95 % CI −0.04 to 0.23, *p* = 0.17; I^2^ = 72.3%, *p* = 0.003) was significantly lower (*p* = 0.001) than that in studies assessing mortality (WMD = 0.54, 95% CI 0.44 to 0.64, *p* < 0.001; I^2^ = 71.7%, *p* = 0.001; Supplementary Figure S2). Furthermore, the pooled WMD in European studies (WMD = 0.52, 95% CI 0.42 to 0.62, *p* < 0.001; I^2^ = 77.1%, *p* ˂ 0.001) was significantly higher (*p* = 0.043) than that in Asian studies (WMD = 0.20, 95% CI 0.04 to 0.37, *p* = 0.014; I^2^ = 82.8%, *p* = 0.001; Supplementary Figure S3).

#### 3.2.6. Certainty of Evidence

The certainty of evidence was downgraded to very low (rating 1) as a consequence of the presence of substantial and unexplained heterogeneity.

### 3.3. Pooled Odds Ratios

#### 3.3.1. Characteristics of Studies

Nine studies in a total of 14,313 COVID-19 patients (49% males, mean age 53 years) reported associations between the De Ritis ratio and disease severity and mortality expressed as ORs in multivariate logistic regression analysis [32,33,46,50,52,55,61,62,63]. Adverse outcomes included mortality (six studies) [32,46,50,52,55,62] and the following measures of severe disease: transfer to ICU (one study) [62], prolonged hospital stay (one study) [62], intubation (one study) [63], persistent viral positivity (one study) [61], and clinical severity according to existing clinical guidelines (one study) [33]. Three studies were conducted in China [32,33,61], three in the USA [55,62,63], one in Austria [46], one in Romania [50], and one in Hungary (Supplementary Table S5) [52].

#### 3.3.2. Risk of Bias

All studies addressed ≥75% of checklist items and consequently were considered as having a low risk of bias (Supplementary Table S4) [32,33,46,50,52,55,61,62,63].

#### 3.3.3. Results of Individual Studies and Syntheses

**Figure 3:**
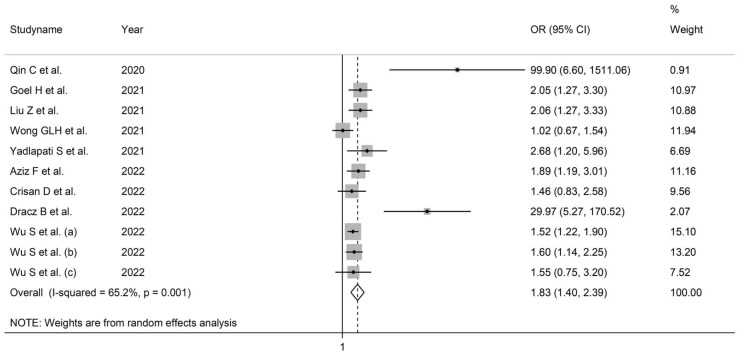
Forest plot of studies examining the association between De Ritis ratio and measures of disease severity or mortality expressed as OR.

Pooled results showed that the De Ritis ratio was significantly associated with the risk of severe disease or death (OR = 1.83, 95% CI 1.40 to 2.39, *p* ˂ 0.001; Figure 3). The substantial between-study heterogeneity observed (I^2^ = 65.2%, *p* = 0.001) warranted random-effects models. The removal of individual studies did not markedly affect the pooled ORs, suggesting the stability of the results (range 1.68–1.96).

#### 3.3.4. Publication Bias

Both the Begg’s (*p* = 0.013) and the Egger’s (*p* = 0.011) tests identified the presence of publication bias. The “trim-and-fill” approach led to the identification of three missing studies that were required as additions to the left side of the funnel plot in order to ensure symmetry (Supplementary Figure S4). The resulting effect size, albeit attenuated, was similar to the primary analysis (OR = 1.60, 95% CI 1.16 to 2.19, *p* = 0.004).

#### 3.3.5. Subgroup and Meta-Regression Analysis

In meta-regression, no significant associations were observed between the OR and age (t = 1.64, *p* = 0.14), the proportion of males (t = 0.42, *p* = 0.68), publication year (t = −0.45, *p* = 0.66), or sample size (t = −1.92, *p* = 0.09). In subgroup analysis, the pooled OR in studies reporting mortality (OR = 2.58, 95% CI 1.42 to 4.68, *p* ˂ 0.001; I^2^ = 73.7%, *p* = 0.002) was non-significantly different (*p* = 0.40) than that in studies reporting severity (OR = 1.56, 95% CI 1.23 to 1.97, *p* ˂ 0.001; I^2^ = 43.4%, *p* = 0.132; Supplementary Figure S5). However, a lower between-study variance was observed in the latter. Similarly, non-significant differences (*p* = 0.59) were observed in pooled ORs between European (OR = 2.84, 95% CI 1.08 to 7.47, *p* = 0.034; I^2^ = 80.9%, *p* = 0.005), Asian (OR = 2.36, 95% CI 0.81 to 6.84, *p* = 0.11; I^2^ = 85.8%, *p* = 0.001), and American studies (OR = 1.63, 95% CI 1.39 to 1.93, *p* ˂ 0.001; I^2^ = 0.0%, *p* = 0.596; Supplementary Figure S6), with a virtual absence of between-study variance in the latter group.

#### 3.3.6. Certainty of Evidence

The certainty of evidence remained low (rating 2) after considering all relevant criteria.

### 3.4. Pooled Hazard Ratios

#### 3.4.1. Study Characteristics

Five studies in a total of 6653 COVID-19 patients (54% males, mean age 62 years) reported associations between the De Ritis ratio and mortality expressed as HRs in multivariate logistic regression analysis [30,33,34,51,54]. Two studies were conducted in China [33,54], one in Israel [30], one in Italy [34], and one in Croatia (Supplementary Table S6) [51].

#### 3.4.2. Risk of Bias

All studies addressed ≥75% of checklist items and hence had a low risk of bias (Supplementary Table S4) [30,33,34,51,54].

#### 3.4.3. Results of Individual Studies and Syntheses

**Figure 4:**
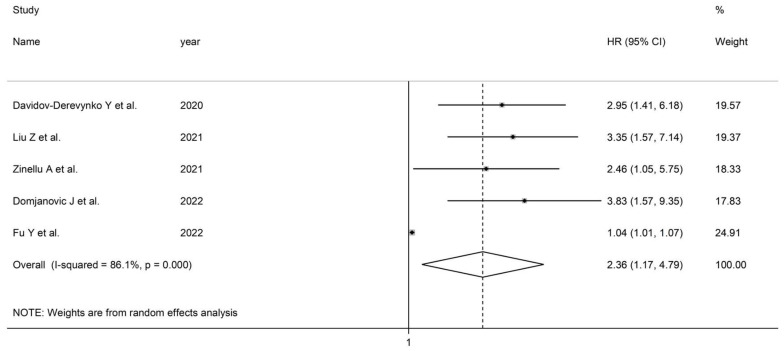
Forest plot of studies examining the association between the De Ritis ratio and measures of disease severity or mortality expressed as HR.

The substantial between-study heterogeneity observed (I^2^ = 86.1%, *p* = 0.001) warranted random-effects models. The De Ritis ratio was significantly higher in non-survivors than survivors (HR = 2.36, 95% CI 1.17 to 4.79, *p* = 0.017; Figure 4). The removal of individual studies did not substantially alter the corresponding pooled HRs, suggesting that the results were stable (range 2.24–3.04).

#### 3.4.4. Publication Bias, Subgroup, and Meta-Regression Analysis

The limited number of studies precluded the assessment of publication bias and subgroup and meta-regression analyses.

#### 3.4.5. Certainty of Evidence

The certainty of evidence was downgraded to extremely low (rating 0) as a consequence of the substantial and unexplained heterogeneity and the lack of assessment of publication bias.

### 3.5. Prognostic Accuracy of the De Ritis Ratio

#### 3.5.1. Characteristics of Studies

Six studies (eight patient groups) in a total of 2069 COVID-19 patients (63% males, mean age 77 years) reported AUROC and cut-off values for the De Ritis ratio in relation to disease severity or mortality [32,34,51,52,56,58]. Endpoints included mortality (five studies) [32,34,51,52,56] and the following measures of severe disease: clinical severity based on existing clinical guidelines (two studies) [56,58] and ICU admission (one study) [56]. One study was conducted in Turkey [56], one in China [32], one in India [58], one in Italy [34], one in Croatia [51], and one in Hungary (Supplementary Table S7) [52].

#### 3.5.2. Risk of Bias

All studies addressed ≥75% of checklist items (Supplementary Table S4) [32,34,51,52,56,58].

#### 3.5.3. Results of Individual Studies and Syntheses

**Figure 5:**
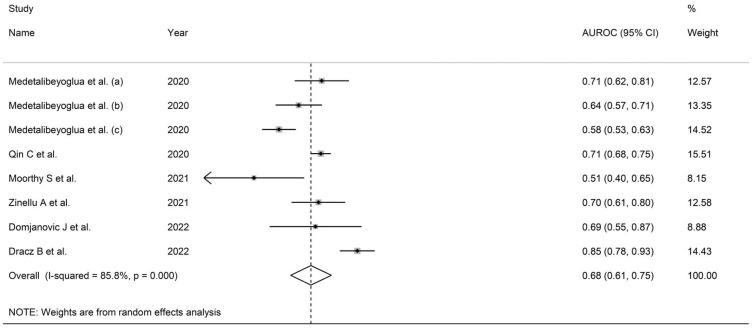
Forest plot of studies investigating the prognostic accuracy of the De Ritis ratio.

The pooled AUROC for the De Ritis ratio was 0.677 (95% CI 0.612 to 0.743, Figure 5). The elevated between-study heterogeneity observed (I^2^ = 85.8%, *p* = 0.001) warranted random-effects models. In sensitivity analysis, the sequential removal of individual studies did not substantially alter the corresponding pooled AUROCs, suggesting that the results were stable (range 0.65–0.70). The cut-off value, sensitivity, and specificity ranges were 1.22–1.65, 0.58–0.74, and 0.56–0.82, respectively (Supplementary Table S7).

#### 3.5.4. Publication Bias

Assessment of publication bias was not possible because of the relatively small number of studies.

#### 3.5.5. Subgroup and Meta-Regression Analysis

In subgroup analysis, the pooled AUROC in studies reporting mortality (AUROC = 0.74, 95% CI 0.68 to 0.81; I^2^ = 71.0%, *p* = 0.008) was significantly higher (*p* = 0.017) than in studies assessing severity (AUROC = 0.59, 95% CI 0.64 to 0.75; I^2^ = 38.5%, *p* = 0.197) (Supplementary Figure S7). Meta-regression analysis could not be performed because of the relatively small number of studies.

## 4. Discussion

In our updated systematic review and meta-analysis, the De Ritis ratio on admission was significantly higher in hospitalized COVID-19 patients with severe disease manifestation or non-survivor status when compared to patients with non-severe disease or survivor status. Such between-group differences were statistically significant regardless of whether they were expressed as WMD, OR, or HR. The capacity of the De Ritis ratio to discriminate between COVID-19 patients with different disease severity and survival status was considered satisfactory, according to a pooled AUROC of 0.677. However, the pooled AUROC was higher (0.74) when considering only studies investigating mortality. Although a moderate–substantial between-study heterogeneity was generally observed, the sequential omission of individual studies did not substantially affect the overall WMD, OR, or HR, suggesting stability of the results. The absence of significant associations between the effect size and a range of clinical characteristics suggests that the De Ritis ratio may offer clinical information that complements, rather than duplicates, the information provided by routine clinical and biochemical parameters in COVID-19. Furthermore, the lack of significant associations between the effect size and the year of study publication indicates that the capacity of the De Ritis ratio to discriminate between patients with different disease severity and survival outcomes is not influenced by temporal changes in the characteristics of the study populations, e.g., different SARS-CoV-2 variants, vaccination type and uptake, and immunomodulatory treatments.

The potential clinical utility of the De Ritis ratio AST/ALT ratio as a biomarker was initially described in 1957 [28]. Whilst the physiological circulating concentrations of ALT and AST represent the equilibrium between hepatocyte turnover and enzyme clearance from serum, their elevations generally indicate the presence of significant hepatocellular damage or death. An increase in serum concentrations of ALT, primarily located in the cytosol of hepatocytes, generally reflects alterations in the cell membrane. By contrast, AST is present not only in the cytoplasm and the mitochondria of hepatocytes but also in the skeletal muscle, heart muscle, brain, kidneys, lungs, pancreas, erythrocytes, and leucocytes, which limits its specificity for liver damage compared to ALT [64,65,66]. The De Ritis ratio has been increasingly studied as a diagnostic and prognostic marker, particularly in acute and chronic viral hepatitis, alcoholic hepatitis, fatty liver, non-alcoholic fatty liver disease, and cirrhosis [29,67,68]. The median and interquartile physiological ranges of the De Ritis ratio have been reported to be 0.90 (0.75–1.10) in healthy females and 0.81 (0.66–1.01) in healthy males [69].

Several mechanisms may account for the onset and progression of liver damage in COVID-19 [16,70,71,72]. For example, SARS-CoV-2 RNA and viral particles have been detected in the liver parenchyma of patients with COVID-19 [73,74,75]. Angiotensin-converting enzyme 2, transmembrane serine protease 2, and paired basic amino acid-cleaving enzyme, critical components for the entry and invasion of host cells by SARS-CoV-2, are expressed in liver cells [76,77,78,79]. Intercellular adhesion molecule-3-grabbing integrin, a liver-specific receptor, CD147, highly expressed in inflammation and infection sites, and antibody-dependent enhancement have also been proposed to facilitate the entry of SARS-CoV-2 in liver cells [80,81,82,83]. Additional potential mechanisms responsible for liver injury in COVID-19 patients include the exposure of the liver parenchyma to high concentrations of circulating pro-inflammatory cytokines [84], the occurrence of hypoxia-reperfusion tissue injury, particularly in the presence of microvascular dysfunction and thrombosis [85], and the occurrence of drug-induced liver injury caused by potentially hepatotoxic medications such as the antiviral remdesivir and the immunomodulating agent tocilizumab [86].

Our meta-analysis provides robust evidence that relatively greater elevations in the De Ritis ratio reflect the presence of more significant structural and functional liver abnormalities, which, in turn, are associated with severe disease and mortality in patients with COVID-19. Therefore, this simply derived biochemical parameter may be particularly useful for early risk stratification and appropriate management in this group. In a systematic review and meta-analysis of eight studies in a total of 4606 patients published in 2021, the De Ritis ratio was significantly higher in COVID-19 patients with poor prognosis, defined as a composite of death, clinical severity, admission to the ICU, and intubation (mean difference = 0.41, 95% CI 0.31 to 0.50, *p* < 0.001). Furthermore, an elevated De Ritis ratio was significantly associated with poor prognosis (OR 3.28, 95% CI 2.39 to 4.52, *p* < 0.001), while the AUC was 0.67 (95% CI 0.63–0.71) [35]. Our updated systematic review and meta-analysis captured a significantly higher number of studies (*n* = 24), which also allowed the conduct of separate sensitivity analyses. Furthermore, we performed subgroup and meta-regression analyses to investigate potential sources of heterogeneity and associations with other variables, including the type of endpoint assessed and the geographical location.

The general moderate–substantial between-study heterogeneity represents a significant limitation of our study, although subgroup analysis identified potential sources of heterogeneity when the effect size was expressed as OR (geographical location and type of endpoint). There was also a significant publication bias in studies reporting the OR, and no assessment could be performed for studies reporting the HR and the AUROC. Furthermore, no study reported long-term, e.g., post-discharge outcomes in COVID-19 patients. By contrast, a significant strength is represented by the wide number of the study geographical locations, which supports the generalizability of our results.

## 5. Conclusions

In our updated systematic review and meta-analysis, higher De Ritis ratios on admission, indicating significant liver injury in the context of excessive systemic inflammation, have been shown to be significantly associated with severe disease and mortality in hospitalized COVID-19 patients. Prospective studies are warranted to determine whether this easily derived parameter of liver function, singly or in combination with other clinical, demographic, or biochemical parameters, can further enhance early risk stratification for short- and long-term outcomes and facilitate treatment strategies to improve clinical outcomes in this group.

## Data Availability

The relevant data are available from A.Z. upon reasonable request.

## Supplementary Materials

The following supporting information can be downloaded at: https://www.mdpi.com/article/10.3390/life13061324/s1, Table S1: PRISMA 2020 for abstracts checklist; Table S2: PRISMA 2020 checklist; Table S3: Studies reporting the De Ritis ratio in COVID-19 patients with different disease severity and survival status; Table S4: The Joanna Briggs Institute critical appraisal checklist; Table S5: Studies reporting the association between the De Ritis ratio and disease severity and survival status in COVID-19 patients using odds ratios; Table S6: Studies reporting the association between the De Ritis ratio and disease severity and survival status in COVID-19 patients using hazard ratios; Table S7: Studies investigating the accuracy of the De Ritis ratio for disease severity or survival status in COVID-19 patients; Figure S1: Funnel plot of studies investigating disease severity and survival status after “trimming-and-filling”. Dummy studies and genuine studies are represented by enclosed circles and free circles, respectively; Figure S2: Forest plot of studies examining the De Ritis ratio in patients with COVID-19 according to disease severity or survival status; Figure S3: Forest plot of studies examining the De Ritis ratio in patients with COVID-19 according to geographical area; Figure S4: Funnel plot of studies investigating the association between the De Ritis ratio and clinical outcomes by means of OR, after “trimming-and-filling”; Figure S5: Forest plot of studies examining the relationship between the De Ritis ratio and clinical outcomes by means of OR, according to measures of disease severity or mortality; Figure S6: Forest plot of studies examining the association between the De Ritis ratio and clinical outcomes by means of OR, according to geographical area; Figure S7: Forest plot of studies investigating the prognostic accuracy of the De Ritis ratio according to measures of disease severity or survival status.
