# Supplementary material for: An Updated Systematic Review and Meta-Analysis of the Association between the De Ritis Ratio and Disease Severity and Mortality in Patients with COVID-19
Source: Life (Basel). 2023 Jun 5;13(6):1324. doi: 10.3390/life13061324 (PMC10303964; doi:10.3390/life13061324)
Supplement: Supplementary file 1 [file life-13-01324-s001.zip › Supplementary_Material_Legends.pdf]

Supplementary material

Supplementary Table 1. PRISMA 2020 for abstracts checklist.

Supplementary Table 2. PRISMA 2020 checklist.

Supplementary Table 3. Studies reporting the De Ritis ratio in COVID-19 patients with different disease severity and survival status.

Supplementary Table 4. The Joanna Briggs Institute critical appraisal checklist.

Supplementary Table 5. Studies reporting the association between the De Ritis ratio and disease severity and survival status in COVID-19 patients using odds ratios.

Supplementary Table 6. Studies reporting the association between the De Ritis ratio and disease severity and survival status in COVID-19 patients using hazard ratios.

Supplementary Table 7. Studies investigating the accuracy of the De Ritis ratio for disease severity or survival status in COVID-19 patients.

Supplementary Figure 1. Funnel plot of studies investigating disease severity and survival status after "trimming-and-filling". Dummy studies and genuine studies are represented by enclosed circles and free circles, respectively.

Supplementary Figure 2. Forest plot of studies examining the De Ritis ratio in patients with COVID-19 according to disease severity or survival status.

Supplementary Figure 3. Forest plot of studies examining the De Ritis ratio in patients with COVID-19 according to geographical area.

Supplementary Figure 4. Funnel plot of studies investigating the association between the De Ritis ratio and clinical outcomes by means of OR, after "trimming-and-filling". Dummy studies and genuine studies are represented by enclosed circles and free circles, respectively.

Supplementary Figure 5. Forest plot of studies examining the association between the Ritis ratio and clinical outcomes by means of OR, according to measures of disease severity or mortality.

Supplementary Figure 6. Forest plot of studies examining the association between the De Ritis ratio and clinical outcomes by means of OR, according to geographical area.

Supplementary Figure 7. Forest plot of studies investigating the prognostic accuracy of the De Ritis ratio according to measures of disease severity or survival status.
