# Supplementary material for: An Updated Systematic Review and Meta-Analysis of the Association between the De Ritis Ratio and Disease Severity and Mortality in Patients with COVID-19
Source: Life (Basel). 2023 Jun 5;13(6):1324. doi: 10.3390/life13061324 (PMC10303964; doi:10.3390/life13061324)
Supplement: Supplementary file 1 [file life-13-01324-s001.zip › Supplementary_Table_3.pdf]

Supplementary Table 3. Studies reporting the De Ritis ratio in COVID-19 patients with different disease severity and survival status.

	Non-severe disease or survivor				Severe disease or non-survivor				
**First author, year, country**	**N**	**Age (Years)**	**Gender (M/F)**	**De Ritis ratio (Mean±SD)**	**N**	**Age (Years)**	**Gender (M/F)**	**De Ritis ratio (Mean ±SD)**	**Outcome**
Davidov-Derevynko Y, 2020, Israel (21)	297	55.8	181/116	1.32±0.53	32	75.5	19/13	1.94±0.87	Mortality
Fu L, 2020, China (44)	211	NR	NR	1.27±0.59	139	NR	NR	1.47±0.67	Severity
Paliogiannis P, 2020, Italy (22)	21	NR	NR	1.27±0.44	9	NR	NR	2.0±0.79	Mortality
An YW, 2021, China (36)	195	41.9	78/117	1.3±0.8	58	56.8	35/23	1.3±0.8	Severity
Benedé-Ubieto R, 2021, Spain (39)	659	NR	NR	1.33±0.04	140	NR	NR	1.8±0.07	Mortality
Chen F, 2021, China (40)	657	46	271/386	1.08±0.6	173	69	107/66	1.31±0.84	Severity
Mittal A, 2021, Nepal (48)	92	35	54/38	1.2±0.44	8	64	6/2	1.6±0.74	Severity
Moorthy S, 2021, India (49)	390	50	233/157	1.72±2.34	66	61	44/22	1.26±1.41	Severity
Ni YN, 2021, China (50)	307	40	135/172	1.01±0.41	526	48	265/261	1.05±0.45	Severity
Ramos-Lopez O, 2021, Mexico (51)	1,645	64	968/677	1.32±0.66	449	76	301/148	1.74±0.76	Mortality/ICU
Zinellu A, 2021, Italy (25)	77	80	27/50	1.27±0.54	28	68	8/20	1.67±0.44	Mortality
Aziz F, 2022, Austria (37)	605	68	423/182	1.28±0.52	142	79	95/47	1.67±0.74	Mortality
Baik SM, 2022, Korea (38)	154	65	74/80	1.6±0.92	49	76	24/25	2.35±1.27	Mortality
Crisan D, 2022, Romania (41)	327	62	190/137	1.2±0.05	43	73	30/13	1.83±0.28	Mortality
Dracz B, 2022, Hungary (43)	286	63	NR	1.0±0.39	36	79	NR	1.8±0.85	Mortality

Legend: M, male; F, female; SD, standard deviation; ICU, intensive care unit; NR, not reported.
