# Supplementary material for: An Updated Systematic Review and Meta-Analysis of the Association between the De Ritis Ratio and Disease Severity and Mortality in Patients with COVID-19
Source: Life (Basel). 2023 Jun 5;13(6):1324. doi: 10.3390/life13061324 (PMC10303964; doi:10.3390/life13061324)
Supplement: Supplementary file 1 [file life-13-01324-s001.zip › Supplementary_Table_4.pdf]

Supplementary Table 4. The Joanna Briggs Institute critical appraisal checklist.

**Study**	**Were inclusion criteria clearly defined?**	**Were the subjects and the setting described in detail?**	**Was the exposure measured in a reliable way?**	**Were objective criteria used for measurement of the condition?**	**Were confounding factors identified?**	**Were strategies to deal with confounding factors stated?**	**Were the outcomes measured in a reliable way?**	**Was appropriate statistical analysis used?**	**Risk of bias**
Davidov-Derevynko Y et al (21)	Yes	Yes	Yes	Yes	Yes	Yes	Yes	Yes	Low
Fu L et al (44)	Yes	Yes	Yes	Yes	Yes	Yes	Yes	Yes	Low
Medetalibeyoglu A et al (47)	Yes	Yes	Yes	Yes	No	No	Yes	Yes	Low
Paliogiannis P et al (22)	Yes	Yes	Yes	Yes	No	No	Yes	Yes	Low
Qin C et al (23)	Yes	Yes	Yes	Yes	Yes	Yes	Yes	Yes	Low
An YW et al (36)	Yes	Yes	Yes	Yes	No	No	Yes	Yes	Low
Benedé-Ubieto R et al (39)	Yes	Yes	Yes	Yes	Yes	Yes	Yes	Yes	Low
Chen F et al (40)	Yes	Yes	Yes	Yes	Yes	Yes	Yes	Yes	Low
Goel H et al (46)	Yes	Yes	Yes	Yes	Yes	Yes	Yes	Yes	Low
Liu Z et al (24)	Yes	Yes	Yes	Yes	Yes	Yes	Yes	Yes	Low
Mittal A et al (48)	Yes	Yes	Yes	Yes	No	No	Yes	Yes	Low
Moorthy S et al (49)	Yes	Yes	Yes	Yes	No	No	Yes	Yes	Low
Ni YN et al (50)	Yes	Yes	Yes	Yes	Yes	Yes	Yes	Yes	Low
Ramos-Lopez O et al (51)	Yes	Yes	Yes	Yes	Yes	Yes	Yes	Yes	Low
Wong GLH et al (52)	Yes	Yes	Yes	Yes	Yes	Yes	Yes	Yes	Low
Yadlapati S et al (54)	Yes	Yes	Yes	Yes	Yes	Yes	Yes	Yes	Low
Zinellu A et al (25)	Yes	Yes	Yes	Yes	Yes	Yes	Yes	Yes	Low
Aziz F et al (37)	Yes	Yes	Yes	Yes	Yes	Yes	Yes	Yes	Low
Baik SM (38)	Yes	Yes	Yes	Yes	Yes	Yes	Yes	Yes	Low
Crisan D et al (41)	Yes	Yes	Yes	Yes	Yes	Yes	Yes	Yes	Low
Domjanović et al (42)	Yes	Yes	Yes	Yes	No	No	Yes	Yes	Low
Drácz et al (43)	Yes	Yes	Yes	Yes	Yes	Yes	Yes	Yes	Low
Fu Y et al (45)	Yes	Yes	Yes	Yes	Yes	Yes	Yes	Yes	Low
Wu S et al (53)	Yes	Yes	Yes	Yes	Yes	Yes	Yes	Yes	Low
