# Supplementary material for: An Updated Systematic Review and Meta-Analysis of the Association between the De Ritis Ratio and Disease Severity and Mortality in Patients with COVID-19
Source: Life (Basel). 2023 Jun 5;13(6):1324. doi: 10.3390/life13061324 (PMC10303964; doi:10.3390/life13061324)
Supplement: Supplementary file 1 [file life-13-01324-s001.zip › Supplementary_Table_5.pdf]

Supplementary Table 5. Studies reporting the association between the De Ritis ratio and disease severity and survival status in COVID-19 patients using odds ratios.

First author, year, country	Study design	N	Age (Years)	Gender (M/F)	OR	(95% CI)	Outcome
Qin C, 2020, China (23)	R	567	55	247/320	99.9	2.1-480.5	Mortality
Goel H, 2021, USA (46)	R	551	64	299/252	2.049	1.273-3.3	Mortality
Liu Z, 2021, China (24)	R	1,788	59	826/913	2.055	1.269-3.327	Severity
Wong GLH, 2021, China (52)	R	7,622	47	3,675/3,947	1.017	0.616-1.418	Persistent positivity
Yadlapati S, 2021, USA (54)	R	200	67	110/90	2.678	1.202-5.963	Intubation
Aziz F, 2022, Austria (37)	R	747	70	518/229	1.89	1.19-3.01	Mortality
Crisan D, 2022, Romania (41)	R	370	66	220/150	1.46	0.71-2.21	Mortality
Dracz B, 2022, Hungary (43)	R	322	66	178/144	29.967	5.266-170.514	Mortality
Wu S (a), 2022, USA (53)	R	2,146	57	944/1,119	1.52	1.22-1.9	Length of stay
Wu S (b), 2022, USA (53)	R	2,146	57	944/1,119	1.6	1.14-2.26	ICU transfer
Wu S (c), 2022, USA (53)	R	2,146	57	944/1,119	1.55	0.75-3.19	Mortality

Legend: M, male; F, female; OR, odds ratio; CI, confidence interval; ICU, intensive care unit; R, retrospective.
