# Supplementary material for: An Updated Systematic Review and Meta-Analysis of the Association between the De Ritis Ratio and Disease Severity and Mortality in Patients with COVID-19
Source: Life (Basel). 2023 Jun 5;13(6):1324. doi: 10.3390/life13061324 (PMC10303964; doi:10.3390/life13061324)
Supplement: Supplementary file 1 [file life-13-01324-s001.zip › Supplementary_Table_6.pdf]

**Supplementary Table 6**. Studies reporting the association between the De Ritis ratio and disease severity and survival status in COVID-19 patients using hazard ratios.

| **First author, year, country** | **Study design** | **N** | **Age (Years)** | **Gender (M/F)** | **OR** | **(95% CI)** | **Outcome** |
|---|---|---|---|---|---|---|---|
| Davidov-Derevynko Y, 2020, Israel (21) | R | 324 | 59 | 232/92 | 2.95 | 1.4-6.15 | Mortality |
| Liu Z, 2021, China (24) | R | 1,788 | 59 | 913/826 | 3.348 | 1.57-7.139 | Mortality |
| Zinellu A, 2021, Italy (25) | R | 105 | 72 | 35/70 | 2.46 | 1.05-5.73 | Mortality |
| Domjanovic J, 2022, Croatia (42) | R | 65 | 57 | 22/43 | 3.83 | 1.57-9.35 | Mortality |
| Fu Y, 2022, China (45) | R | 4,371 | 64 | NR | 1.04 | 1.01-1.07 | Mortality |

Legend: M, male; F, female; NR, not reported; OR, odds ratio; CI, confidence interval; R, retrospective.
