# Supplementary material for: An Updated Systematic Review and Meta-Analysis of the Association between the De Ritis Ratio and Disease Severity and Mortality in Patients with COVID-19
Source: Life (Basel). 2023 Jun 5;13(6):1324. doi: 10.3390/life13061324 (PMC10303964; doi:10.3390/life13061324)
Supplement: Supplementary file 1 [file life-13-01324-s001.zip › Supplementary_Table_7.pdf]

Supplementary Table 7. Studies investigating the accuracy of the De Ritis ratio for disease severity or survival status in COVID-19 patients.

First author, year, country	Study design	N	AUC (95% CI)	Cut-off	Sensitivity	Specificity	Outcome
Medetalibeyoglu A (a), 2020, Turkey (47)	R	554	0.713 (0.618-0.807)	1.65	0.575	0.823	Mortality
Medetalibeyoglu A (b), 2020, Turkey (47)	R	554	0.636 (0.564-0.709)	1.26	0.649	0.604	ICU
Medetalibeyoglu A (c), 2020, Turkey (47)	R	554	0.577 (0.529-0.625)	1.55	0.61	0.556	Severity
Qin C, 2020, China (23)	R	567	0.71 (0.67-0.74)	1.38	NR	NR	Mortality
Moorthy S, 2021, India (49)	R	456	0.51 (0.42-0.69)	NR	NR	NR	Severity
Zinellu A, 2021, Italy (25)	R	105	0.701 (0.603-0.787)	1.49	0.74	0.60	Mortality
Domjanovic J, 2022, Croatia (42)	R	65	0.691 (0.537-0.845)	1.48	0.58	0.72	Mortality
Dracz B, 2022, Hungary (43)	R	322	0.85 (0.777-0.923)	1.218	0.72	0.75	Mortality

Legend: AYC, area under the curve; ICU, intensive care unit; NR, not reported; R, retrospective.
