# Supplementary figures and images for: An Updated Systematic Review and Meta-Analysis of the Association between the De Ritis Ratio and Disease Severity and Mortality in Patients with COVID-19
Source: Life (Basel). 2023 Jun 5;13(6):1324. doi: 10.3390/life13061324 (PMC10303964; doi:10.3390/life13061324)

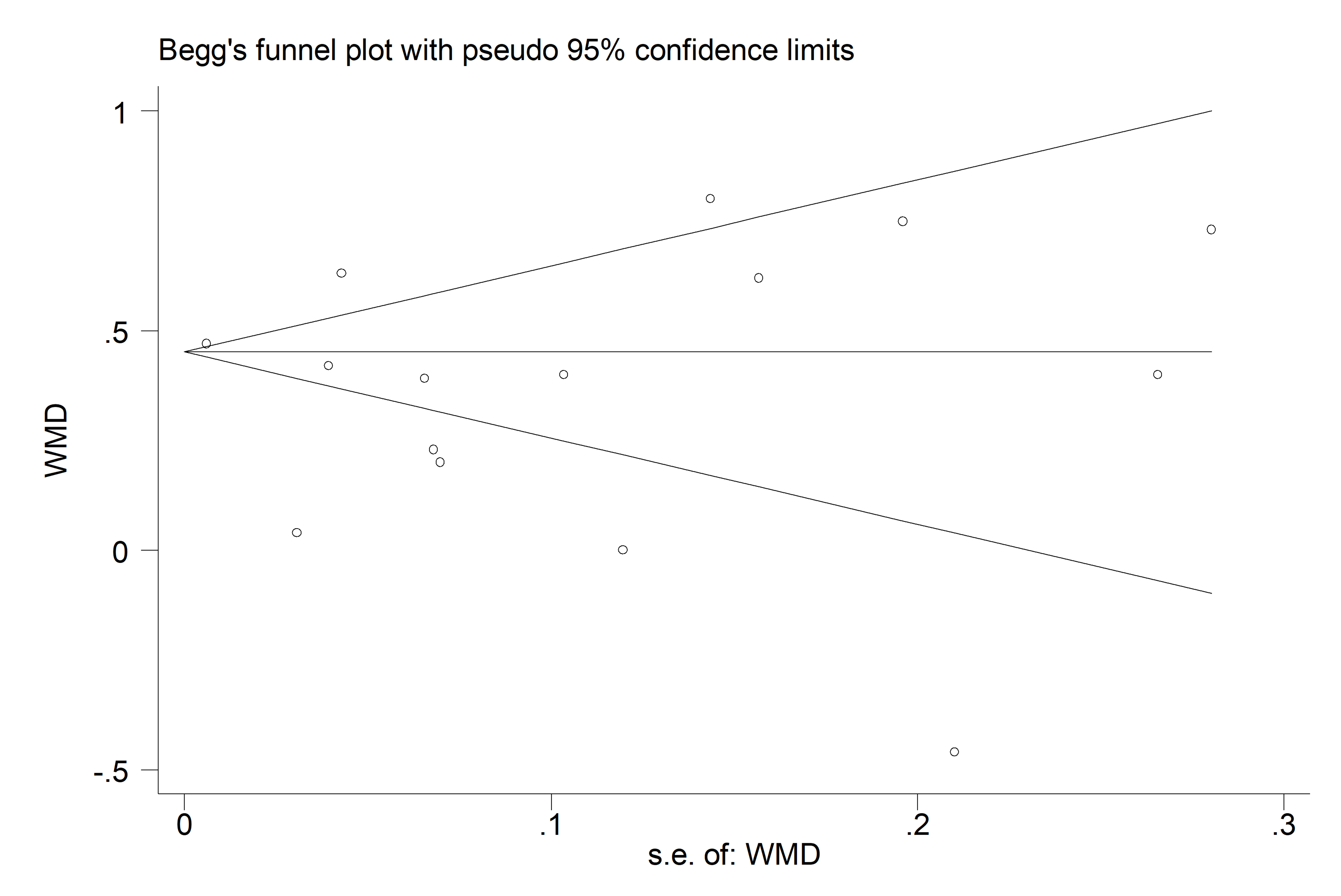

Supplement: Supplementary file 1 [file life-13-01324-s001.zip › Supplementary_Figure_1.tif]

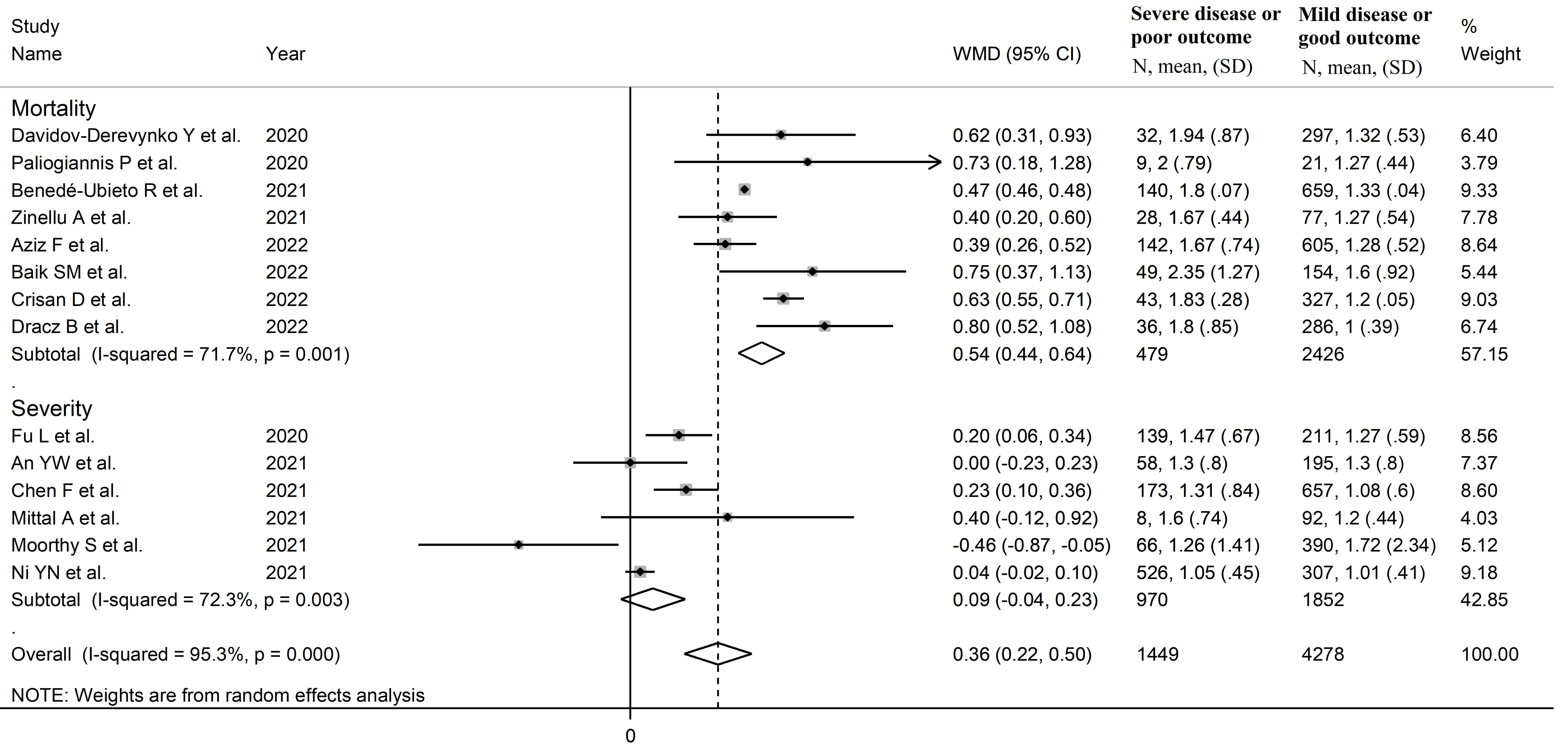

Supplement: Supplementary file 1 [file life-13-01324-s001.zip › Supplementary_Figure_2.tif]

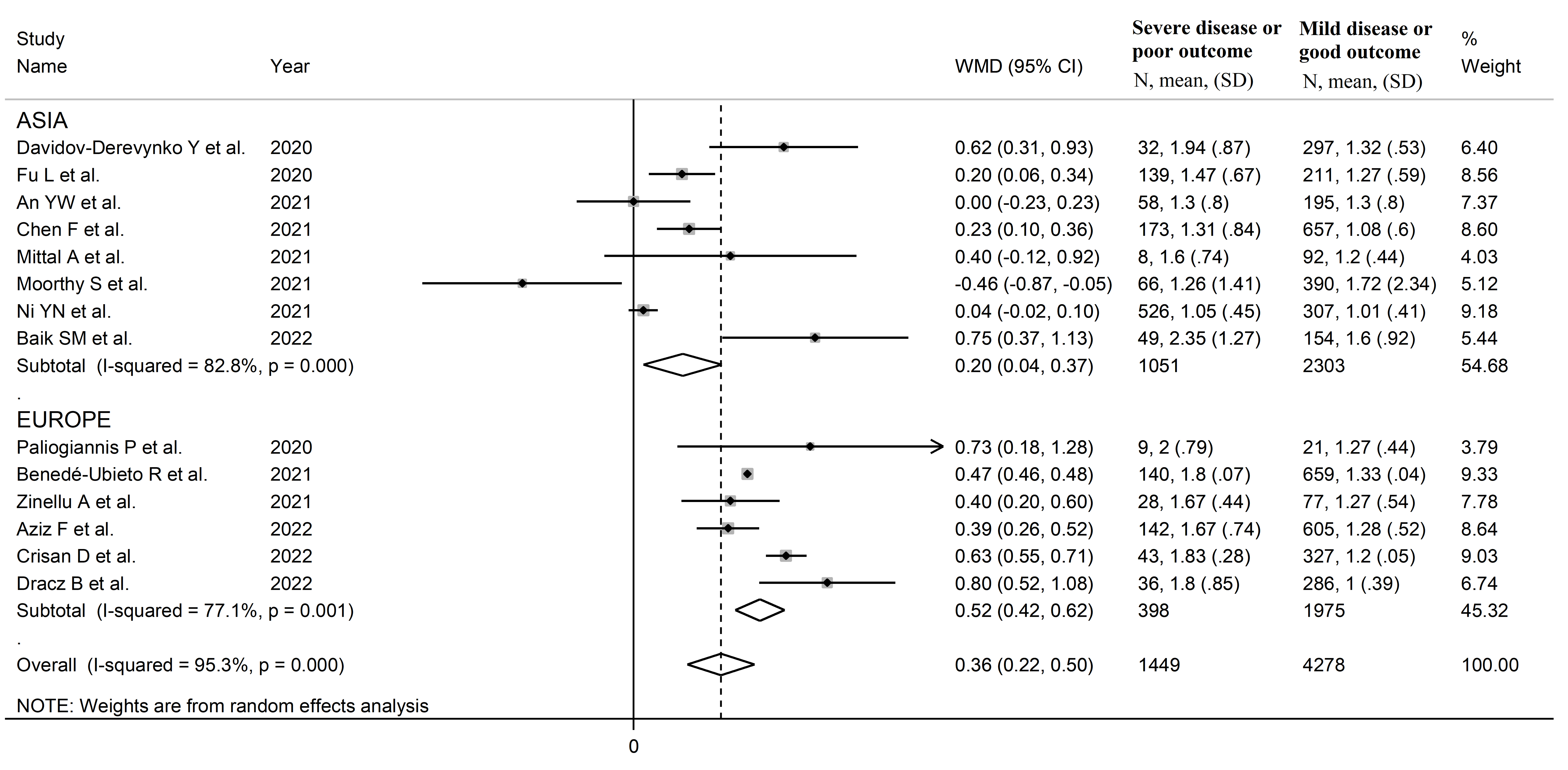

Supplement: Supplementary file 1 [file life-13-01324-s001.zip › Supplementary_Figure_3.tif]

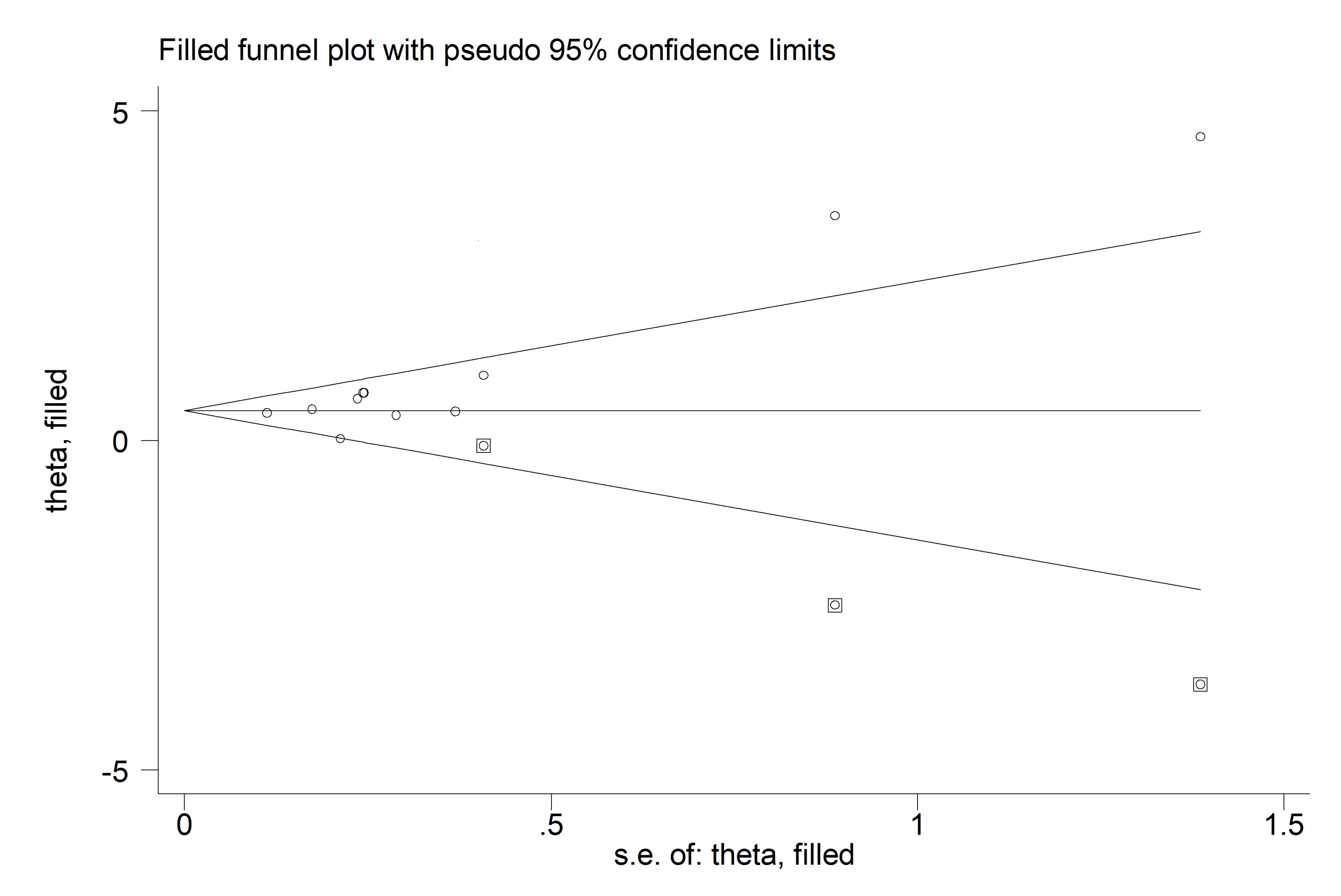

Supplement: Supplementary file 1 [file life-13-01324-s001.zip › Supplementary_Figure_4.tif]

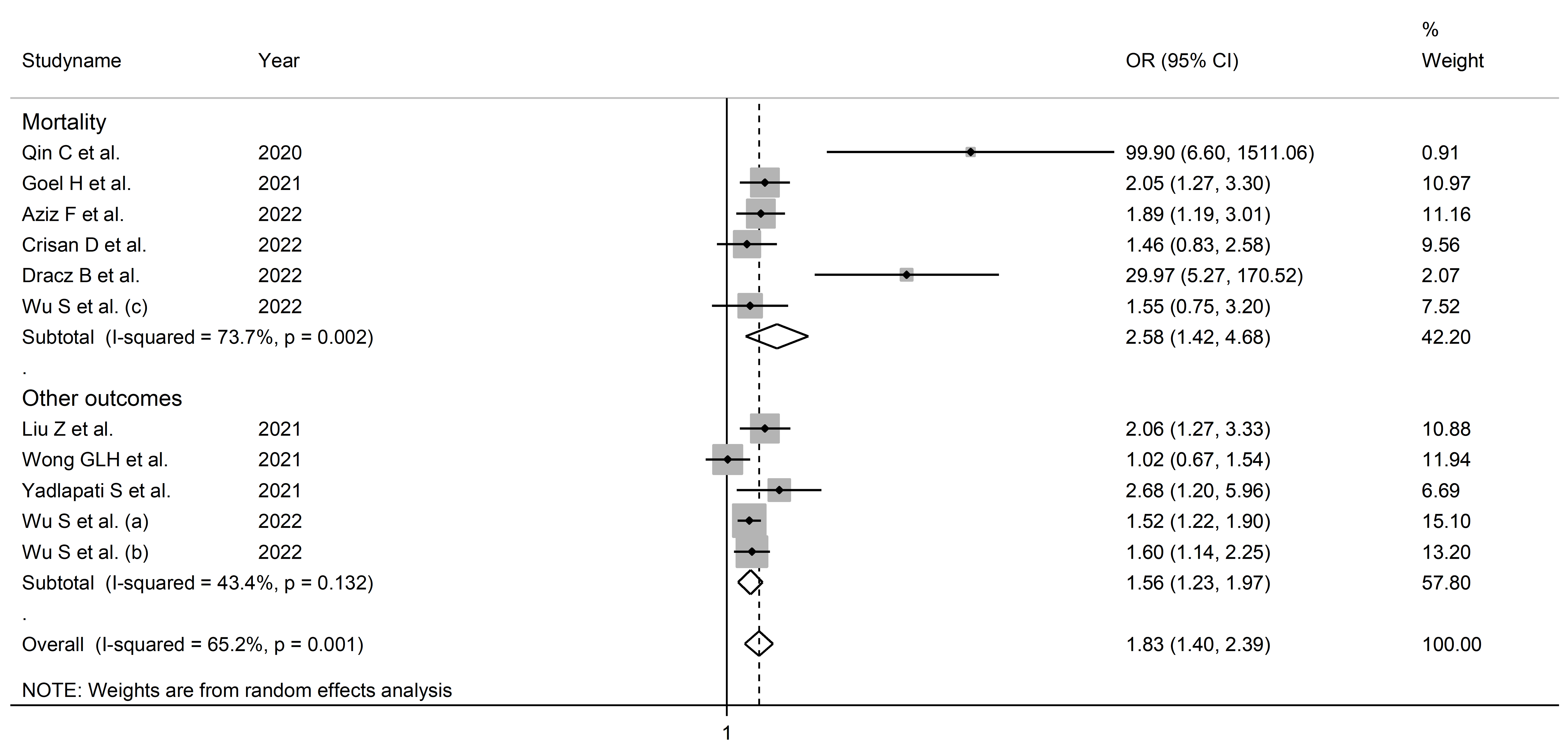

Supplement: Supplementary file 1 [file life-13-01324-s001.zip › Supplementary_Figure_5.tif]

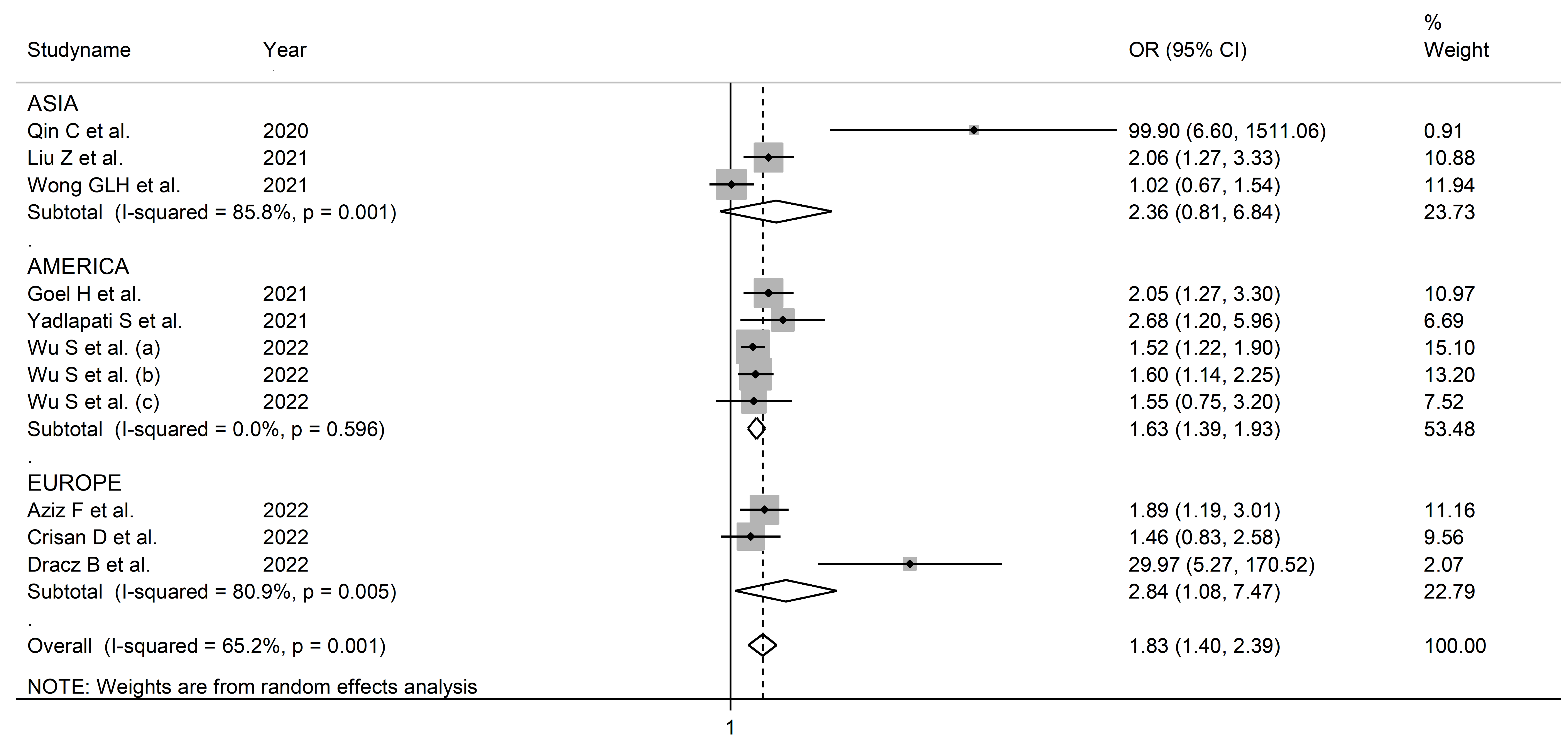

Supplement: Supplementary file 1 [file life-13-01324-s001.zip › Supplementary_Figure_6.tif]

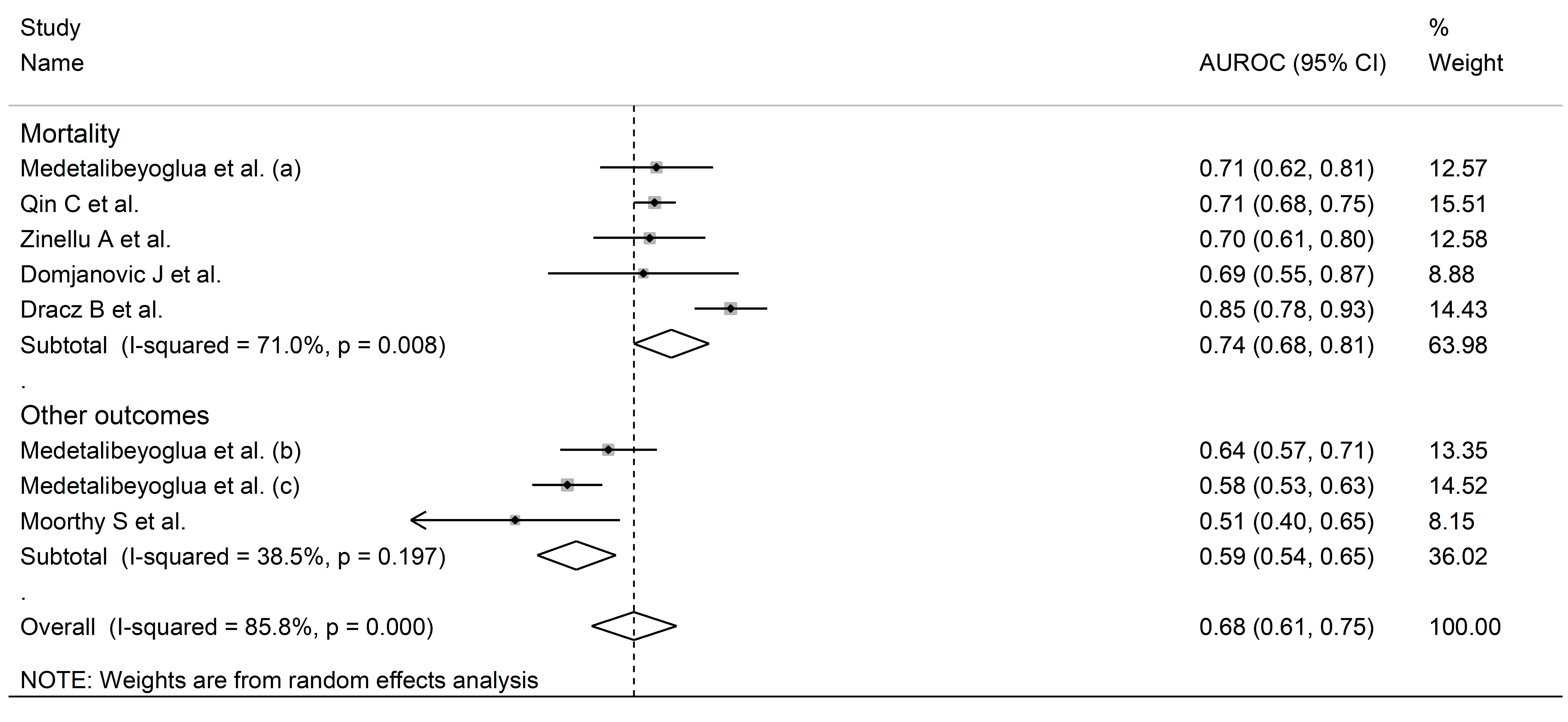

Supplement: Supplementary file 1 [file life-13-01324-s001.zip › Supplementary_Figure_7.tif]
